# Case Report: Nephrotic syndrome caused by mercury poisoning due to freckle-removing cream

**DOI:** 10.3389/fmed.2025.1651441

**Published:** 2025-11-07

**Authors:** Can Wu, Dandan Liu

**Affiliations:** 1 Department of Pulmonary and Critical Care Medicine, Anhui No. 2 Provincial People’s Hospital, Hefei, Anhui, China; 2 Anhui Province Key Laboratory of Occupational Health, Anhui No. 2 Provincial People’s Hospital, Hefei, Anhui, China

**Keywords:** membranous nephropathy, mercury poisoning, nephrotic syndrome, chelation therapy, cosmetics

## Abstract

Nephrotic syndrome caused by mercury poisoning is rare and can often be misdiagnosed. We report a 33-years-old female patient who experienced fatigue and edema of the lower extremities for 1 month. A renal biopsy confirmed the diagnosis of membranous nephropathy (MN) for the patient. Her urinary mercury level was measured at 56.22 ug/L, with a reference range of 0–10 ug/L. A cream used to remove freckles was identified to contain about 13276 mg/kg mercury, which is the possible mercury source. The patient’s symptoms improved after chelation therapy, and their urinary mercury levels gradually decreased. After 12 months of follow-up, the patient remained in a state of complete remission. Using mercury-based cosmetics over an extended period can lead to mercury poisoning and trigger MN. Chelation therapy serves as an effective treatment, yielding positive clinical outcomes.

## 1 Introduction

As a toxic heavy metal, mercury silently exists in human daily life and work. Prolonged or high levels of mercury exposure can cause mercury poisoning. Common sources of mercury exposure include occupational settings, broken thermometers, mercury-containing folk remedies, and cosmetics (1). When mercury accumulates in the human body, it can cause damage to various systems, including the skin, gastrointestinal tract, kidneys, and neuromuscular system (2). Diagnosing mercury poisoning can be difficult due to its unusual clinical symptoms and often overlooked history of exposure. This report outlines the clinical characteristics, diagnosis, treatment, and prognosis of a patient with nephrotic syndrome (NS) resulting from mercury poisoning caused by a facial cream containing mercury.

## 2 Case presentation

A 33-years-old woman visited the outpatient clinic, presenting with a 1-month history of fatigue and edema in both legs. She had no fever, reduced urine output, gross hematuria, and flank pain. Physical examination showed moderate pitting edema in both lower extremities. She had a 3-year history of hypothyroidism, for which she regularly took levothyroxine sodium. The patient denied having hypertension, cardiovascular disorder, chronic kidney disease, or diabetes mellitus. Upon examination, urine tests showed a positive protein (+++) result. The serum total protein (TP) was measured at 44.9 g/L, and the albumin (ALB) level was 24.4 g/L. The renal function was normal.

Upon admission, further examinations were performed. Daily blood pressure and temperature monitoring were conducted, yielding normal results. Color Doppler ultrasound examination was conducted for the heart, kidneys, and veins of the lower extremities, which showed no abnormalities. Both the electrocardiograph and chest X-ray results were normal. Laboratory tests indicated: 24-h urine protein at 5.8 g, total cholesterol (TC) at 6.03 mmol/L, low-density lipoprotein cholesterol (LDL-C) at 3.29 mmol/L, serum calcium at 1.94 mmol/L, hemoglobin (Hb) at 104 g/L, and red blood cell count (RBC) at 3.71 × 10∧12/L. The patient’s fasting blood glucose, thyroid function, immunoglobulin (Ig) levels, coagulation function, and complement C3/C4 levels tests were normal. Viral tests for hepatitis B and C, as well as HIV, were negative. Serum tests for anti-M-type phospholipase A2 receptor (PLA2R), antineutrophil cytoplasmic antibody (ANCA), antidouble-stranded DNA (dsDNA), and antinuclear antibody (ANA) all returned within normal limits.

**FIGURE 1:**
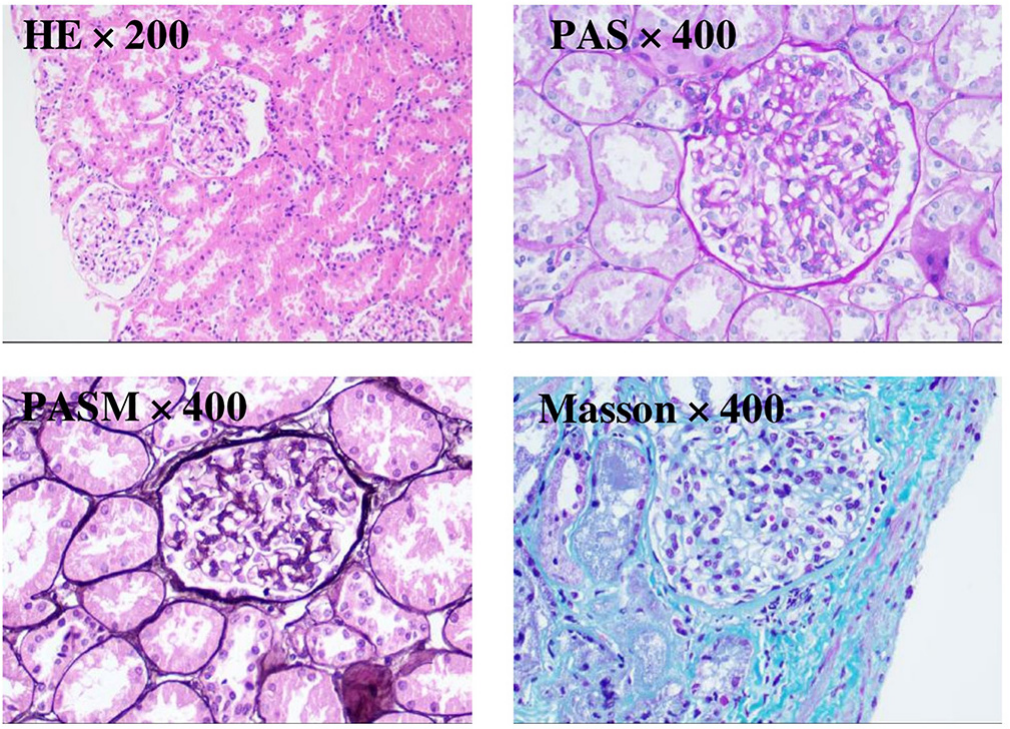
Renal biopsy was examined under light microscopy with HE staining (×200), PAS staining (×400), PASM staining (×400), and Masson staining (×400).

**FIGURE 2:**
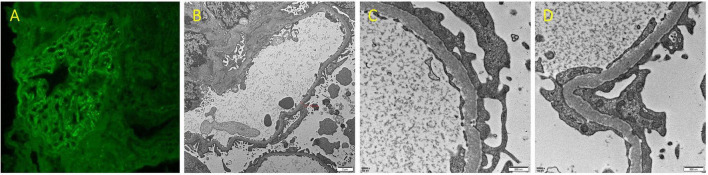
**(A)** Immunofluorescence staining for IgG of renal biopsy (×400). **(B–D)** Renal biopsy under electron microscopy (**B** ×6000; **C,D** ×30000).

In view of NS, a renal biopsy was performed. Light microscopy revealed a stiff glomerular capillary loop, mild segmental proliferation of mesangial matrix and cells, and subepithelial fuchsinophilic deposits along the glomerular basement membrane (Figure 1). The immunofluorescence (IF) showed diffuse, granular IgG deposits in the glomerular capillary loop (Figure 2A). IF staining for PLA2R, thrombospondin type 1 domain containing 7A (THSD7A), and neural epidermal growth factor-like 1 protein (NELL-1) was negative. Electron microscopy demonstrated diffuse fusion (>80%) of podocyte foot processes, with small amounts of electron-dense deposits subepithelially (Figures 2B–D). These findings are consistent with membranous nephropathy (MN).

No evidence was found to suggest that diabetes mellitus, systemic lupus erythematosus (SLE), or infection, which are known to lead to secondary MN, were present. Therefore, the possibility of heavy metal poisoning was considered. Urine tests revealed a urinary mercury level of 56.22 ug/L, exceeding the reference range of 0–10 ug/L. To determine the source of mercury, the patient was thoroughly questioned. She was a housewife and denied using any folk remedies. Additionally, none of her family members worked in industries related to mercury. Further investigation showed that she had been using a freckle-removing cream purchased online for 6 months, but stopped using it upon her admission. Testing revealed that the level of mercury in the cream was 13276 mg/kg, significantly exceeding the Chinese National Standards of 1 mg/kg or lower.

**FIGURE 3:**
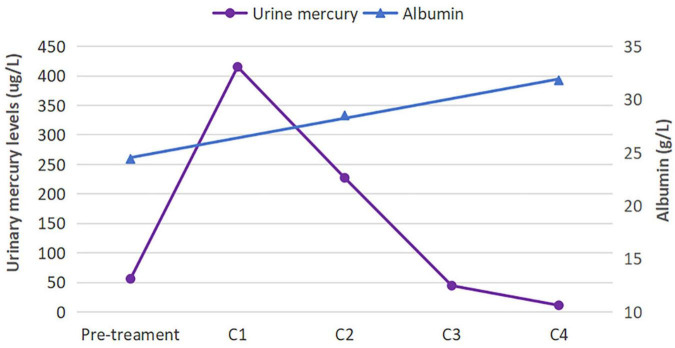
Urinary mercury and albumin levels before and after treatment. The urinary mercury level initially rose before rapidly declining, whereas the albumin level showed a steady rise. C, course of chelation therapy.

Given the evidence of mercury poisoning, the patient underwent chelation therapy with a 0.25 g intramuscular injection of sodium dimercaptopropane sulfonate (DMPS), once a day for 3 days, followed by intermittent treatment for 4 days. Additional treatments included irbesartan at a dosage of 75 mg daily and calcium supplements. After undergoing four courses of chelation therapy, her symptoms of fatigue disappeared, and the edema was relieved. Her urinary mercury level declined to 14.52 ug/L, and ALB increased to 31.8 g/L. During a 12-months outpatient follow-up, the levels of urinary mercury, urine protein, and ALB returned to normal, and the patient reported no discomfort. Figure 3 illustrates the changes in urinary mercury and ALB levels.

## 3 Discussion

Nephrotic syndrome is not challenging to diagnose clinically, as it exhibits characteristics such as significant proteinuria, hypoproteinemia, edema, and hyperlipidemia. The emphasis lies on differentiating between primary and secondary NS, which is crucial for determining subsequent treatment options. In this case, the patient was diagnosed with MN through a renal biopsy. The blood test and IF staining for PLA2R were negative, supporting the secondary MN diagnosis (3). After ruling out potential causes, such as diabetes mellitus and SLE, mercury poisoning was eventually identified as the underlying issue. Following four courses of chelation therapy, the patient’s symptoms improved, and complete remission was achieved during the 12-months follow-up period.

Mercury exists in three forms: elemental, organic, and inorganic. Common sources of human exposure to elemental mercury include thermometers, dental amalgam, and gold mining activities. The primary route of mercury entry into the human body is through inhaling mercury vapor (4). In the environment, methylmercury is the predominant form of organic mercury. Prolonged and excessive consumption of contaminated fish can lead to the accumulation of methylmercury in the body, resulting in mercury poisoning (5). Inorganic mercury compounds are used in the production of catalysts, antiseptics, and pigments. Mercury sulfide (HgS) has been incorporated into some traditional and folk remedies for medical use for thousands of years. *In vivo*, inorganic mercury ions can bind with sulfhydryl groups of tyrosinase, inhibiting its activity and reducing melanin production (6). Due to this property, inorganic mercury is added to some whitening cosmetics, which is illegal.

Mercury poisoning from cosmetics has become a global health concern. Despite international bans, mercury-containing cosmetics are still available online and in stores. The public uses these products for skin whitening, but often overlooks their safety. Several reports on mercury poisoning have shown that the mercury content in these cosmetics exceeds the limit significantly (7, 8), as in this case. Table 1 summarizes the characteristics of reported cases of mercury-induced NS. Notably, the most common source of mercury was whitening cosmetics (16 out of 24 cases). Even children experienced mercury poisoning through indirect contact with facial creams containing mercury (8, 9). As shown in Table 1, cosmetic products primarily affected women, especially young women, which aligns with the literature (1). In China, mercury poisoning must be diagnosed and treated at designated medical institutions like the Institute of Occupational Disease Prevention and Control. Consequently, most clinicians have limited experience with diagnosing and treating this condition, which can lead to misdiagnosis. Therefore, clinicians should consider the possibility of mercury exposure when they encounter a young female with unexplained NS.

**TABLE 1:** Clinical characteristics of cases of mercury-induced nephrotic syndrome.

Cases	Age/sex (number of cases)	Source of mercury	Urine mercury (reference value)	Renal pathology (number of cases)	Treatment	Outcome
1. Liu et al. (20)	55/M	Thermometer	>500 (0–2 μmol/mmol Cr)	MN	Chelation therapy with DMPS	CR
2. Qin et al. (21)	73/M	Ointment for dermatitis	33.4 (<15 ng/ml)	NA	Chelation therapy with DMPS	PR
3. Ji et al. (22)	33/M 30/F	Skin-lightening cream	65.4; 80 (<4 μg/g Cr)	MN (1) NA (1)	Chelation therapy with DMPS	NA
4. Jawandhiya et al. (23)	26/F 22/F	Fairness facial cream	29.71; NA (0.14–4.2 ug/L)	MN (2)	Modified Ponticelli regimen (alternating months of steroids and cyclophosphamide)	CR
5. Gao et al. (24)	65/F	Skin lightening cream	27.5 (<4 μg/g creatinine)	MCD with IgA deposition	Chelation therapy with DMPS	CR
6. Pathak et al. (25)	47/F	Siddha medicine	17.7 (<10 microgram/liter)	MN	Angiotensin receptor blockers	PR
7. Yawei et al. (26)	44/F	Hair dyes + skin lightening cream	122.5 (<8 μg/day)	MN	Chelation therapy with DMSA + prednisone + FK506	PR
8. Onwuzuligbo et al. (27)	14/M	Mercury vapor in the environment	42.9 (≤20 μg/d)	MN	Chelation therapy with DMSA + prednisolone	NA
9. Niu et al. (28)	39/F	Skin lightening cream	90 (<4 μg/g creatinine)	MCD with IgA nephropathy	Chelation therapy with DMPS + medrol	CR
10. Zhang et al. (29)	28/F	Skin lightening cream	469 (<50 μmol/L)	MCD	Chelation therapy with DMPS + prednisone	CR
11. Wagrowska-Danilewicz et al. (30)	42/M	Self-injection of elemental mercury	830 μg/L	MCD	Chelation therapy with DMPS + steroids	CR
12. Tang et al. (31)	26–45/F (4)	Skin lightening cream	316–2521 (<35 nmol/d)	MCD (3) MCD with IgA nephropathy (1)	2 cases received chelation therapy with D-penicillamine; 2 cases received chelation therapy with D-penicillamine + steroids	CR
13. Miller et al. (32)	60/M	Contaminated fish	39 (<20 nmol/d)	FSGS	Prednisone	Death
14. Saleem et al. (33)	62/M	Gold amalgam extraction	2519 (level of concern, >5.8 nmol/mmol)	MCD	Chelation therapy with DMSA + prednisolone	CR
15. Chakera et al. (34)	26–44/F (2)	Skin lightening cream	16.5; 77.5 (<5.5 nmol/mmol creatinine)	MN (2)	Stopping use of the cream	NA
16. Campbell et al. (35)	25/M	Lighting tubes	127.5 (<5.5 nmol/mmol creatinine)	MCD	Chelation therapy with DMSA + prednisolone	CR
17. Tang et al. (36)	34/F	Skin lightening cream	287 (<50 nmol/L)	MCD	Chelation therapy with D-penicillamine	CR
18. This case	33/F	Freckle-removing cream	56.22 (0–10 ug/L)	MN	Chelation therapy with DMPS	CR

MN, membranous nephropathy; MCD, minimal change disease; FSGS, focal segmental glomerulosclerosis; DMPS, sodium dimercaptopropane sulfonate; DMSA, dimercaptosuccinic acid; CR, complete remission; PR, partial remission; NA, not available.

The clinical signs of mercury poisoning affect multiple systems. Most cases show neurological symptoms such as headache, dizziness, limb numbness, tremor, neuromuscular pain, fatigue, insomnia, and memory decline. Kidney damage mainly presents as edema, foamy urine, nephrotic syndrome, and hematuria. Gastrointestinal symptoms include abdominal pain, decreased appetite, nausea, vomiting, and oral ulcers. Skin lesions include rash and peeling (1, 2). Inhaling mercury vapor may cause pneumonitis, cough, chest pain, and dyspnea, potentially leading to respiratory failure (10). Since these symptoms are non-specific, patients are often misdiagnosed at their initial visit.

The pathological types of mercury-associated NS in Table 1 included MN (10/22), minimal change disease (MCD) (11/22), and focal segmental glomerulosclerosis (FSGS) (1/11). MN and MCD were the most common pathological patterns, which were consistent with the literature (11, 12). However, the exact mechanism of mercury-related kidney damage is not fully understood. High concentrations of mercury directly harm renal tubular epithelial cells, leading to tubular necrosis through the induction of oxidative stress (13). Immune mechanisms play a crucial role in mercury-related kidney damage. Mercury stimulates the production of immunoglobulin antibodies (IgG1, IgG2a, IgG3, and IgM) in autoimmune-prone mice, and granular deposits of these circulating antibodies are observed in the renal mesangium (14). IgG1 and IgG2a antibodies eluted from the glomeruli of mercury-exposed rats were pathogenic and triggered MN in unexposed rats following intravenous injection (15). In this case, renal biopsy revealed granular deposits of IgG along the glomerular capillary loop. These findings suggest that mercury-induced immune activation causes the formation of *in situ* immune complexes, leading to glomerular lesions.

The most important step in treating mercury poisoning is to eliminate the source of exposure. Additionally, chelation therapy, glucocorticoids, and immunosuppressants are available options (Table 1). For symptomatic patients, chelating agents such as DMPS and dimercaptosuccinic acid (DMSA) are commonly used treatments. Chelation therapy is unnecessary for asymptomatic patients. In these cases, mercury levels will gradually decrease after removing the source, without any complaints (8, 16). Therefore, the decision to initiate chelation therapy relies on a thorough clinical assessment. Another question is whether glucocorticoids are necessary for mercury-associated glomerular diseases or not. There were no significant differences in treatment outcomes for patients with mercury-associated NS between those treated with DMPS alone and those who received DMPS in combination with glucocorticoids. In severe cases, glucocorticoids may be used concurrently (12). Our patient was cured after solely receiving DMPS, which helped avoid potential side effects associated with glucocorticoids. Although hemodialysis was ineffective at removing mercury even with chelation therapy before renal function recovery, it was necessary for renal support in patients who developed acute renal failure (17). In contrast, plasma exchange can effectively eliminate mercury and enhance the patient’s condition. For acute or severe mercury poisoning cases, plasma exchange might be considered (18, 19).

In summary, mercury poisoning is uncommon but treatable. Recognizing it is crucial to prevent misdiagnosis and unsuitable treatment. This report emphasizes the significance of thoroughly investigating the patient’s history during the diagnostic process. Additionally, chelation therapy serves as an effective treatment option.

## Data Availability

The original contributions presented in this study are included in this article/Supplementary material, further inquiries can be directed to the corresponding author.
